# Effects of Titanium Dioxide Nanoparticles on Photosynthetic and Antioxidative Processes of *Scenedesmus obliquus*

**DOI:** 10.3390/plants9121748

**Published:** 2020-12-10

**Authors:** Zhou Li, Philippe Juneau, Yingli Lian, Wei Zhang, Shanquan Wang, Cheng Wang, Longfei Shu, Qingyun Yan, Zhili He, Kui Xu

**Affiliations:** 1 Environmental Microbiomics Research Center, School of Environmental Science and Engineering, Southern Marine Science and Engineering Guangdong Laboratory (Zhuhai), Sun Yat-sen University, Guangzhou 510006, Guangdong, China; lizh255@mail2.sysu.edu.cn (Z.L.); lianyli@mail.sysu.edu.cn (Y.L.); zhangwei26@mail.sysu.edu.cn (W.Z.); wangshanquan@mail.sysu.edu.cn (S.W.); wangcheng5@mail.sysu.edu.cn (C.W.); shulf@mail.sysu.edu.cn (L.S.); yanqy6@mail.sysu.edu.cn (Q.Y.); 2 Department of Biological Sciences, GRIL-EcotoQ-TOXEN, Ecotoxicology of Aquatic Microorganisms Laboratory, Université du Québec à Montréal, Succ. Centre-Ville, Montréal, QC H3C 3P8, Canada; juneau.philippe@uqam.ca; 3 College of Agronomy, Hunan Agricultural University, Changsha 410128, Hunan, China

**Keywords:** titanium dioxide nanoparticle, photosynthesis, oxidative stress, *Scenedesmus obliquus*

## Abstract

The effects of the photocatalytic toxicity of titanium dioxide nanoparticle (nano-TiO_2_) on phytoplankton are well understood. However, as UV light intensity decreases sharply with the depth of the water column, the effects of nano-TiO_2_ itself on deeper water phytoplankton, such as green algae, need further research. In this research, we investigated the effects of three sizes of TiO_2_ (10, 50 and 200 nm) on the photosynthetic and antioxidative processes of *Scenedesmus obliquus* in the absence of UV light. We found that 50 nm and 10 nm TiO_2_ (10 mg/L) inhibited growth rates and the maximal photosystem II quantum yield compared to the control in *Scenedesmus obliquus*. The minimal and maximal fluorescence yields, and the contents of reactive oxygen species and lipid peroxidation, increased, indicating that photosynthetic energy/electrons transferred to oxygen and induced oxidative stress in nano-TiO_2_-treated samples. In addition, we found that aggregations of algae and 10 nm TiO_2_ were present, which could induce cell membrane disruption, and vacuoles were induced to cope with nano-TiO_2_ stress in *Scenedesmus obliquus*. These results enhance our understanding of the effects of nano-TiO_2_ on the photosynthetic and antioxidative processes of green algae, and provide basic information for evaluating the ecotoxicity of nano-TiO_2_ in freshwater ecosystems.

## 1. Introduction

Since TiO_2_ nanoparticles (nano-TiO_2_) are widely used in commercial and industrial fields [1,2], they are inevitably released into freshwater ecosystems [3,4]. In freshwater ecosystems, it has been found that the concentration of nano-TiO_2_ ranges from 0.2 µg/L to 16 µg/L [5,6,7], and its modeled concentrations in waste water treatment plant effluents could reach up to 3 mg/L, and this is expected to dramatically increase in natural ecosystems in the future [8,9,10]. As abundant small (around 0.6–200 µm) single or clustered cells with high surface-to-volume ratios suspended in freshwater ecosystems [11], phytoplankton have a high probability of encountering suspended particles, such as TiO_2_ nanoparticles. The nano-TiO_2_ pollution in freshwater ecosystems could bring adverse effects on these photosynthetic organisms due to its aggregation with phytoplankton and/or its photocatalytic effects. Phytoplankton are the dominant primary producers in freshwater ecosystems [12], and thus they are the base of the aquatic food web and an important component of the carbon cycle, as well as other biogeochemical cycles [13,14]. Therefore, a better understanding of the toxicological effects and underlying mechanisms of TiO_2_ nanoparticles on phytoplankton is essential.

The photocatalytic activity of nano-TiO_2_ illuminated by UV light can damage algae by directly reacting with cell membranes or generating reactive oxygen species (ROS) [14], which is accepted as one of the main toxicity mechanisms of nano-TiO_2_ on phytoplankton [15,16,17]. However, some phytoplankton, such as green algae, are mainly found at depths greater than one meter [18,19], where UV intensity is less than 1% of that on the surface water in many lakes, such as Taihu Lake in China [20,21]. This low UV intensity is unlikely to activate nano-TiO_2_ particles and induce the strong photocatalytic inhibition of phytoplankton. Therefore, photocatalytic inhibition should not be the main toxicity mechanism of nano-TiO_2_ for algae in deeper waters. On the other hand, studies of nano-TiO_2_ toxicity on green algae without UV can provide the information needed to better understand the ecotoxicological effects of nano-TiO_2_ itself on freshwater ecosystems. However, these toxic effects on green algae are still uncertain and require further investigation.

The toxicity of nano-TiO_2_ could also be due to its aggregation with phytoplankton and subsequent influence on light absorbance and nutrient uptake [22,23]. Moreover, the aggregation between phytoplankton and TiO_2_ nanoparticles could also result in phytoplankton cell membrane damage by increasing the permeability or disrupting the cell membrane [24]. This cell membrane damage was shown to induce a series of metabolic changes in phytoplankton. Indeed, phytoplankton could induce exopolysaccharide (EPS) production to cope with nano-TiO_2_’s adverse effects by decreasing the direct interaction between nanoparticles and cell membrane [25,26]. If nanoparticles enter into the cell by permeating the cell membrane, they may react with organelles and therefore compromise their functions. It has been found that TiO_2_ nanoparticles could alter the chloroplast functions of dinoflagellate *Karenia brevis*, and therefore inhibit photosynthesis [22].

Photosynthesis inhibition could result in reactive oxygen species (ROS) production generated by the energy transfer from triplet chlorophyll molecules to oxygen or electrons transfer to oxygen [27,28]. As one of the main ROS, singlet oxygen species could react with the photosystem II (PSII) reaction center D1 protein and degrade it, causing PSII inhibition [29]. Mitochondria are another ROS production site in plant cells [30], but scarce information exists about the toxicological effects of nano-TiO_2_ on respiration in phytoplankton. In order to prevent or cope with ROS, phytoplankton may trigger protection mechanisms, such as non-photochemical quenching (NPQ) and increasing antioxidant enzymatic activity [31]. However, we still lack information about how nano-TiO_2_ induces photosynthetic and antioxidative protection mechanisms and interacts with green algae in freshwater ecosystems.

In this research, we used *Scenedesmus obliquus*, a model species for studying the toxicological effects of pollutants [26,32,33], to investigate the toxicological effect of nano-TiO_2_ on the photosynthetic and antioxidative processes under artificial fluorescent light without UV illumination, in order to obtain a better understanding of the mode of action of these particles. If the cell membrane, photosynthesis and respiration were affected, we would determine the inhibitory targets of nano-TiO_2_, and then how and which, if any, response mechanisms would be triggered to cope with this stress condition. We found that nano-TiO_2_ could directly disrupt the algal cell membrane, and inhibit the oxygen evolution and PSII activity of *S. obliquus,* while it induced a series of response mechanisms to cope with these stress conditions.

## 2. Results

### 2.1. The Effects of TiO_2_ on S. obliquus Growth

**Figure 1:**
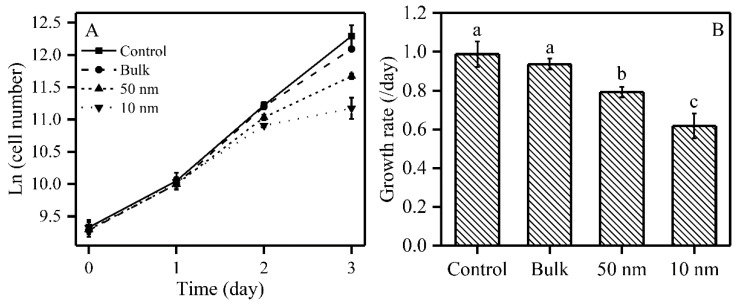
Effects of three sizes of TiO_2_ particles on the growth of *S. obliquus*. Panel (**A**): Growth curves; Panel (**B**): Growth rate; Different letters on the error bars represent significant (Tukey’s honestly significant difference (HSD), *p* < 0.05) differences. Values are represented as means ± SD (*n* = 6).

In order to explore the effect of TiO_2_ (10 mg/L) on the growth of *S. obliquus*, cell numbers were measured daily and growth rates were calculated during a 72 h TiO_2_ treatment. The effect of three sizes of TiO_2_ on the growth of *S. obliquus* is shown in Figure 1. Two sizes of nano-TiO_2_ inhibited the growth on the second day, and this inhibition was enhanced on the third day in *S. obliquus* (Figure 1A). The 72 h growth rate was therefore reduced in the presence of TiO_2_ particles; the growth rate was significantly inhibited by 20% when exposed to 50 nm TiO_2_, while 10 nm TiO_2_ inhibited the growth rate strongly by 38%. However, the bulk (200 nm) TiO_2_ did not induce significant effects on the growth rate for the studied species.

### 2.2. Photosynthesis and Respiration of S. obliquus under TiO_2_ Treatments

**Figure 2:**
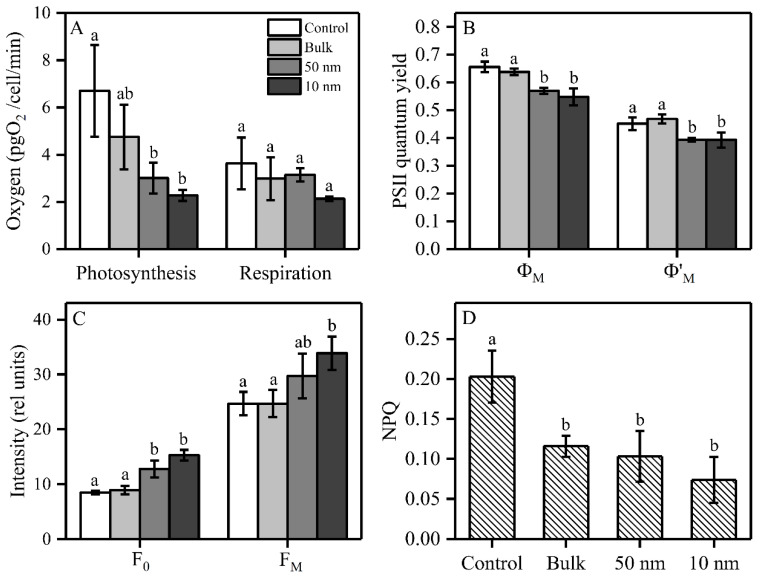
Effects of three sizes of TiO_2_ on photosynthetic parameters in *S. obliquus* after 72 h exposure. (**A**) Oxygen evolution/consumption under growth light and dark conditions, respectively; (**B**) PSII quantum yield; (**C**) Maximal and minimal fluorescence yields (F_M_ and F_0_); and (**D**) non-photochemical quenching (NPQ). Different letters on the error bars represent significant (Tukey’s HSD, *p* < 0.05) differences. Values are represented as means ± SD (*n* = 3).

In order to examine the effect of TiO_2_ on the photosynthesis and respiration of *S. obliquus*, we measured the oxygen evolution, oxygen consumption and chlorophyll induction curves under three sizes of TiO_2_ after 72 h exposure (Figure 2). The photosynthetic oxygen evolution was significantly inhibited by 55% and 66% under 50 and 10 nm TiO_2_, respectively, while respiration was inhibited by 41% under 10 nm TiO_2_, but without significance (Figure 2A). The maximal PSII quantum yield (Φ_M_) was significantly inhibited by 13% and 17% under 50 and 10 nm TiO_2_, respectively, compared to the control, while there was no significant difference between these two sizes of nano-TiO_2_. A similar tendency was found for the operational PSII quantum yield (Φ’_M_), which was significantly inhibited by 13% under both 50 and 10 nm TiO_2_, but bulk TiO_2_ did not significantly inhibit Φ_M_ and Φ’_M_ (Figure 2B). Both 50 nm and 10 nm TiO_2_ significantly increased F_0_ by respectively 51% and 80%, while there was no significant difference in F_0_ between these two sizes of nano-TiO_2_. For F_M_, only the 10 nm TiO_2_ significantly increased by 37% compared to the control (Figure 2C). The three sizes of TiO_2_ (200, 50 and 10 nm) significantly decreased NPQ by 43%, 49% and 63%, respectively, while there was no significant difference in NPQ among the three sizes of TiO_2_ (Figure 2D)_._ In order to explore the photosynthetic responses to TiO_2_ over the entire exposure time, we also measured the induction curves of the studied species after 24 h and 48 h, to obtain the PSII quantum yields, NPQ and relative electron transport rate (rETR) (Supplementary Materials Figure S1). The Φ_M_, Φ’_M_ and rETR were not inhibited after 24 h, but showed an inhibition at 48 h and 72 h for two sizes of nano-TiO_2_ (50 nm and 10 nm) (Figure S1A–C,G–I). On the other hand, NPQ was stimulated for the 10 nm TiO_2_-treated samples at 24 h and 48 h compared to control, but was sharply decreased at 72 h of exposure (Figure S1D–F).

### 2.3. Oxidative Process of S. obliquus under TiO_2_ Treatments

**Figure 3:**
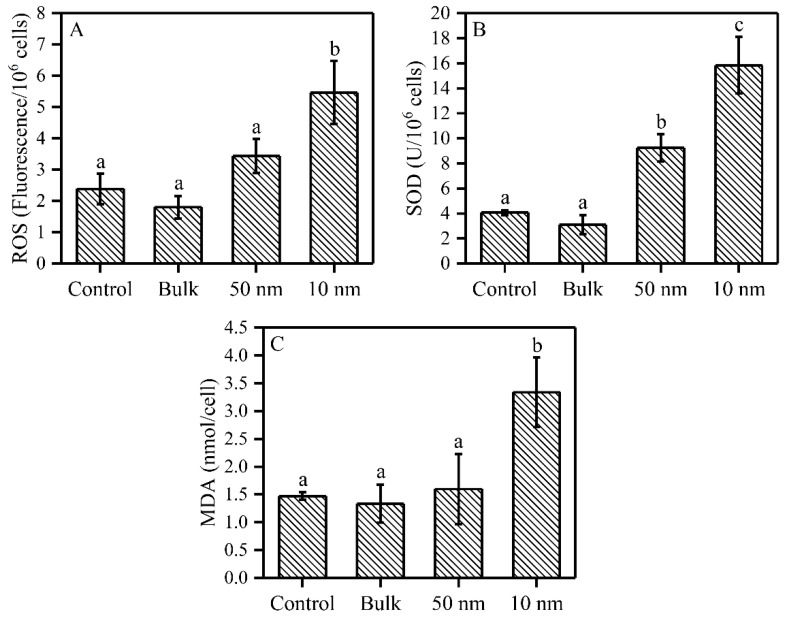
ROS, SOD and MDA contents of *S. obliquus* under three sizes of TiO_2_ exposure after 72 h. (**A**) ROS content; (**B**) SOD activity; and (**C**) MDA content. Different letters on the error bars show significant (Tukey’s HSD, *p* < 0.05) differences. Values are represented as means ± SD (*n* = 3).

The effects of three sizes of TiO_2_ on ROS, SOD and MDA content, after 72 h exposure, are shown in Figure 3. The 10 nm TiO_2_ significantly increased the intracellular ROS content by 129% compared to control, while the 50 nm and bulk TiO_2_ did not have such an effect compared to the control samples (Figure 3A). Both nano-TiO_2_ particles significantly increased the intracellular SOD activity by 128% (50 nm) and 290% (10 nm) (Figure 3B). Similarly to ROS, MDA content was only significantly increased by 127% in the presence of the 10 nm TiO_2_ particles compared to the control (Figure 3C).

### 2.4. Pigments of S. obliquus under TiO_2_ Treatments

Both nano-TiO_2_ sizes (50 and 10 nm) caused a significant decrease in Chl *a* concentration (respectively by 56% and 63%) after 72 h exposure compared to the control samples (Table 1). However, the three tested sizes of TiO_2_ did not significantly affect Chl *b* and total carotenoids in the studied species. It was found that 50 nm TiO_2_ significantly increased the Chl *b*/Chl *a* ratio by 55% compared to the control cells. We also noticed that 50 and 10 nm TiO_2_ significantly increased the ratio of Car/Chl *a* by 33% and 50%, respectively (Table 1).

### 2.5. Aggregation between Algae and Three Sizes of TiO_2_

**Figure 4:**
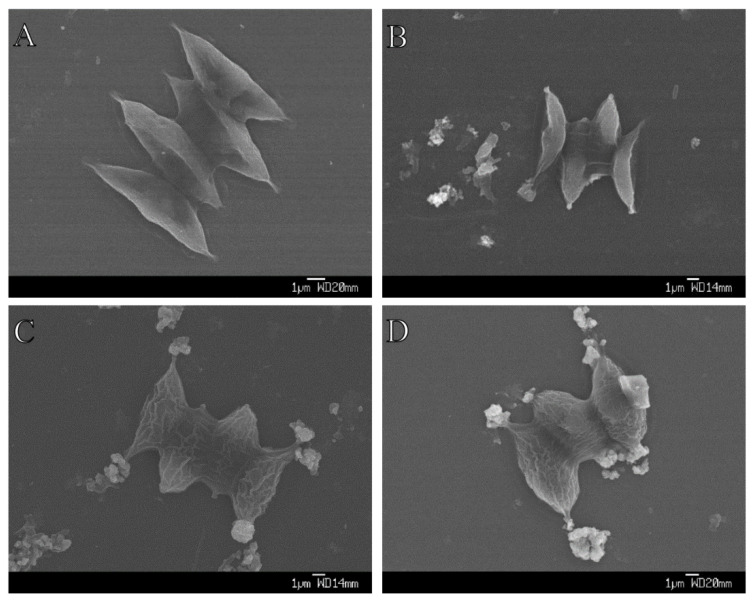
Aggregation between *S. obliquus* and three sizes of TiO_2_ after 72 h exposure. (**A**) Control; (**B**) Bulk TiO_2_; (**C**) 50 nm TiO_2_; and (**D**) 10 nm-TiO_2_.

**Figure 5:**
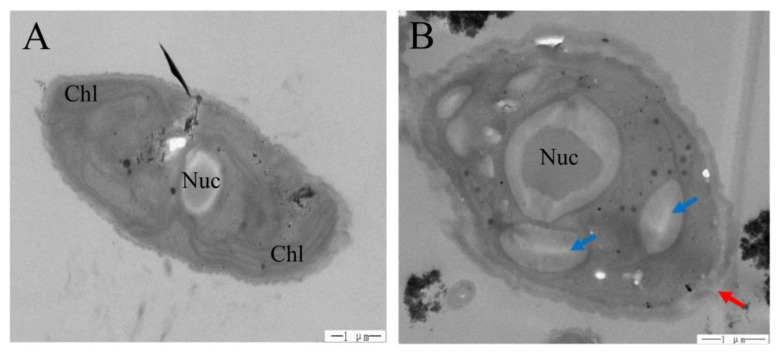
Effects of 10 nm TiO_2_ on the cell structure of *S. obliquus* after 72 h exposure. (**A**) Control, and (**B**) 10 nm-TiO_2_. Blue arrow shows a vacuole while red arrow shows cell membrane breaking. Chl: Chloroplast; Nuc: Nucleus.

We found that the aggregation between algae and TiO_2_ was more obvious as the size of TiO_2_ decreased (Figure 4). Moreover, we clearly saw that wrinkles were induced outside the algal cell under both nano-TiO_2_ samples (Figure 4C,D), but not in the bulk TiO_2_ samples (Figure 4B). The damaging effects of 10 nm TiO_2_ on algal cells and the intracellular structure were largely due to the disruption of cell membranes (Figure 5A), and impaired chloroplast and vacuoles were clearly induced under this nano-TiO_2_ size (Figure 5B).

## 3. Discussion

The concentrations of nano-TiO_2_ that were effective on the growth of green alga range widely from 0.2 to 250 mg/L, according to previous published papers. These variations in the observed effects are mainly caused by the different experimental designs, the sizes of nano-TiO_2_, and the studied species [24,34,35,36,37,38]. However, most of those experiments were performed under artificial fluorescent light that emits some UV radiations [14]. In this research, we clearly demonstrated that both the size fractions of nano-TiO_2_ (10 mg/L) inhibited the growth and photosynthesis of *S. obliquus*. Moreover, bulk TiO_2_ did not inhibit growth at the concentration of 10 mg/L, suggesting that there is no toxicity induced by bulk TiO_2_ for the studied species below this concentration. Therefore, the inhibition of growth in the studied species induced by nano-TiO_2_ seems to be mainly caused by the particle size instead of the TiO_2_ properties.

When we compare the impacts of different sizes of TiO_2_, 10 nm nano-TiO_2_ induced a stronger growth inhibition compared to 50 nm nano-TiO_2_ in *S. obliquus*, but the impact on photosynthesis (Φ’_M_) was not different between the two sizes. These different effects between growth and photosynthesis suggest that other metabolic mechanisms than photosynthesis could have been involved when the algae were exposed to 10 nm nano-TiO_2_ compared to 50 nm nano-TiO_2_. We also demonstrated that the F_0_ increased without a significant change of F_M_ (although an increasing trend was observed) under 50 nm nano-TiO_2_ treatment, while both F_0_ and F_M_ increased under 10 nm nano-TiO_2_ treatment in the studied species. It has been suggested that an increase in F_0_ without obvious changes in F_M_ indicates a blockage of the energy transfer among antenna pigments (and/or from antenna pigments to reactions centers) in cyanobacteria exposed to zinc [39]. We can therefore advance that the 50 nm nano-TiO_2_ induced mainly a similar impact on energy transfer, while under the 10 nm nano-TiO_2_ both energy transfer inhibition and a damaged electron transport chain were observed.

Due to the induced inhibition of PSII quantum yields and oxygen production, we can expect that the excess energy would be dissipated through non-photochemical quenching (NPQ) mechanisms, as shown previously [40,41,42]. Surprisingly, it is interesting to note that NPQ decreased under the two sizes of nano-TiO_2_ treatment, which indicates that NPQ is not an induced mechanism to cope with TiO_2_ stress conditions in *S. obliquus*. This decreased NPQ may result from the decreased Chl *a* content, which prevents a strong excitation pression at PSII, and also the damaged chloroplast structure (Figure 5B) necessary for the induction of NPQ [43,44]. Consistently, our recent study found that NPQ was not induced under stress conditions (mesotrione exposure) for another green alga *Chlamydomonas reinhardtii* either [45]. Therefore, these results suggest that NPQ may not be the main response mechanisms to pollutants (e.g., nano-TiO_2_ or metals) in green algae [39].

Energy transfer among antenna and the blockage of photosynthetic electron transfer could result in excess energy accumulation [28]. As mentioned, this excess energy should normally be dissipated through NPQ; otherwise, excess energy could form triplet chlorophylls that transfer energy to oxygen to produce singlet oxygen [46]. This increased singlet oxygen in theory could therefore contribute to the increase in ROS under nano-TiO_2_ treatment, but this needs to be further explored. On the other hand, the relative electron transport rate inhibited under nano-TiO_2_ treatment (Figure S1I) may also lead to the forming of ROS when electrons react with oxygen, and therefore also contribute to the increase in ROS. Superoxide, as an important ROS, may be generated through Mehler reaction at the PSI level, resulting in oxygen consumption. The increased SOD activity detected for the nano-TiO_2_-treated samples suggests an increased Mehler reaction activity, and therefore a higher superoxide production. Those increased ROS could compete with oxygen evolution at PSII, which can explain why the oxygen evolution was much more inhibited than the PSII quantum yield under nano-TiO_2_ treatment. Moreover, respiration also seems to be inhibited by this particle size, although not significantly, which suggests that respiration may also contribute to ROS production and subsequently induce cell membrane lipid peroxidation under 10 nm nano-TiO_2_. Additionally, an increased ROS content could damage the thylakoid membranes and the structure of the light-harvesting complexes [47]. The increased SOD activity observed in the 50 nm nano-TiO_2_ treatment may also explain the small damage caused by ROS in these treated cells, while the scavenging system was not strong enough to scavenge the ROS produced under 10 nm nano-TiO_2_. Therefore, the decreased energy transfer from light-harvesting complexes to reaction centers and/or electron transfer after PSII should occur, leading to an increased production of ROS due to the energy accumulation at the light-harvesting complexes and/or the inhibition of electron transfer.

We clearly demonstrated that aggregation between algae and nano-TiO_2_ was more abundant as the size of TiO_2_ decreased, as was also seen with nano and bulk ZnO in *Phaeodactylum tricornutum* [48]. This aggregation was suggested to be blocking light absorption and inhibiting algal growth, a phenomenon called the shading effect [35,36]. NPQ decreased under bulk TiO_2_, which may suggest that the photosynthetic energy absorption efficiency was increased, which implied a shading effect under bulk TiO_2_ treatment samples. However, growth rate, F_0_ and chlorophyll content were not affected in the samples exposed to bulk TiO_2_, suggesting that the shading effect was not induced, at least not obviously, by bulk and nano-TiO_2_ particles. Therefore, aggregation between algae and the nano-TiO_2_ should induce other adverse effects on algae than the shading effect. Indeed, from the TEM results, we found that 10 nm TiO_2_ disrupted algal cell membranes and induced holes in algal cell membranes. Similar results were also induced by nano-ZnO in another green alga, *Pseudokirchneriella subcapitata* [37]. These broken cell membranes may allow the entrance of nano-TiO_2_ particles into the algal cell and induce more damages. One of the most obvious damages induced by nano-TiO_2_ was the destruction of thylakoid membrane stability, which was demonstrated by the loss of intact chloroplasts, and this is in agreement with previous studies showing the chloroplast impairment/degradation induced by nano-TiO_2_ in the green alga *Chlorella pyrenoidosa* and the higher plant *Arabidopsis thaliana* [49,50]. Therefore, this damage could impact the structural pigment–protein complexes and finally inhibit the energy transfer from light-harvesting complexes to reaction centers, which is also suggested by the chlorophyll fluorescence yield changes (F_0_ and F_M_) as discussed above. We therefore propose that the light-harvesting complex may be one of the main photosynthetic apparatus targets of nano-TiO_2_ in the studied species. In order to more efficiently cope with intracellular nano-TiO_2_, the studied species induced vacuoles to isolate nano-TiO_2_ and protect the thylakoid membrane. This protection mechanism (vacuole accumulation) was shown to be sufficient to isolate metals (such as Cu and Cd), and therefore to play an important role under metal stress conditions in green algae and higher plants [51,52].

Considering that the growth rate, the maximal PSII quantum yield and the NPQ were not significantly affected by TiO_2_ exposure in the first 24 h, and that the inhibition of growth and photosynthesis was gradually observed from 24 to 72 h (Figure S1), we speculate that nano-TiO_2_ particles firstly disrupt cell membranes and thylakoid membranes, then impact the structural pigment–protein complexes, and inhibit light energy transfer from light-harvesting complexes to reaction centers and photosynthetic electron transport rate, then finally induce oxidative stress and growth inhibition in *S. obliquus*.

In this study, we revealed the effects of the toxicity and protection mechanisms of nano-TiO_2_ on membrane stability, photosynthetic energy transfer processes and anti-oxidative processes in *S. obliquus*. We clearly demonstrated the possible inhibition of these particles, at high concentrations, of the growth and photosynthesis of freshwater green algae found in deeper waters, and therefore the present study provides new insights into our understanding of the toxicity of nano-TiO_2_ on phytoplankton. Although the current environmental concentrations of nano-TiO_2_ do not reach the concentration used in the present study, these findings are essential to better understand what will happen eventually, since water concentrations of nano-TiO_2_ are expected to dramatically increase in the near future [53].

## 4. Materials and Methods

### 4.1. Algae and Nano-TiO_2_

*Scenedesmus obliquus* FACHB416 (hereafter *S. obliquus*) was obtained from the Freshwater Algae Culture Collection of the Institute of Hydrobiology, the Chinese Academy of Sciences. This alga was grown in a 250 mL flask containing 150 mL BG11 medium [54] placed on a constant rotary shaker (100 rpm), which was in a growth chamber under a 14:10 h light/dark cycle with a light intensity of 150 µmol photons m^−2^∙s^−1^ and 24 °C. In order to avoid the UV emission from artificial fluorescence light, light came sideways into the flasks and a thick glass was placed between the fluorescence light tubes and flasks. We confirmed that the UV (290–390 nm) intensity was zero inside the flasks by a UV light meter (UV−340A, Lutron, Taipei, China). Cells were kept in their exponential growth phase and cell count was determined using a Multisizer^TM^ 3 Coulter Counter^®^ (Beckman Coulter Inc., Brea, CA, USA). The growth rate was calculated as µ = (ln(A3) − ln(A0))/3, in which A3 and A0 are the cell numbers of samples at the start day and the third day, respectively.

Anatase nano-TiO_2_ and bulk TiO_2_ (three different average sizes: 10, 50 and 200 nm) were purchased from Shanghai Aladdin Biochemical Technology CO., Ltd., Shanghai, China. The size and properties of nano-TiO_2_ and bulk TiO_2_ were confirmed by SEM (details in 2.4) and X-ray Diffraction (XRD; Ultima IV, Rigaku Corporation, Tokyo, Japan). Stock solutions of nano-TiO_2_ and bulk TiO_2_ (10,000 mg/L) were prepared with sterile MQ water and were kept at 4 °C until use. Nano-TiO_2_ and bulk TiO_2_ (equal volume of sterile MQ water for control) were sonicated for 30 min and then added to the cultures with a final nominal concentration of 10 mg/L, which was the lowest concentration inducing effects on the studied species. The original cell concentration in each flask was approximately 1 × 10^4^ cells/mL. After adding the three size fractions of TiO_2_ to the cultures, the cells were grown under the above conditions for 72 h (usual duration for studying toxicity of nano-TiO_2_ on phytoplankton for short-term exposure).

### 4.2. Oxygen Evolution/Consumption and Chlorophyll a Fluorescence Measurement

After 72 h exposure, oxygen evolution/consumption under growth light/dark was measured by a YSI 5100 Dissolved Oxygen Meter (YSI, Yellow Springs, OH, USA) and the concentrations of samples were kept the same at around 2 × 10^5^ cells/mL. A fluorescence induction curve was measured by a WATER-PAM Chlorophyll Fluorometer (Walz GmbH, Effeltrich, Germany) according to Ref. [55]. In brief, cultures were dark-acclimated for 15 min, and then minimal fluorescence yield (F_0_) and maximal fluorescence yield (F_M_) were measured respectively before and after being pulsed with a saturating light (800 ms, 3000 µmol photons m^−2^∙s^−1^). After that, an actinic light with the same intensity as the growth light was turned on for 6.5 min, and every half minute a saturating light was applied to get the fluorescence yield F’ and F’_M_ before and after the saturating light pulse. Maximal PSII quantum yield (Φ_M_) and operational PSII quantum yield (Φ’_M_) were calculated following these equations: Φ_M_ = (F_M_ − F_0_)/F_M_ [56] and Φ’_M_ = (F’_M_ − F’)/F’_M_ [57]. Non-photochemical quenching was calculated as: NPQ = (F_M_ − F’_M_)/F’_M_ [58]. NPQ and Φ’_M_ are the average values calculated from the last three saturating light measurements.

### 4.3. ROS, SOD and MDA Measurements

2’-7’-Dichlorofluorescin Diacetate (DCFH-DA) was purchased from Shanghai Aladdin Biochemical Technology CO., Ltd., China and used to measure reactive oxygen species in the studied algae following the instruction of the manufacturer. In brief, after 72 h TiO_2_ treatment, 10 mL of samples were collected by centrifugation (8000× *g*) for 10 min. Two ml DCFH-DA were added to the pellet and incubated at 37 °C for 30 min, then 2 mL fresh PBS buffer was used to wash the samples three times to remove the DCFH-DA in the solution. At last, the 522 nm fluorescence emission was recorded by exciting the samples at 488 nm with a microplate reader (Thermo Fisher, Waltham, MA, USA).

SOD activity and the MDA content were determined by using two different reagent kits (Nanjing Jiancheng Biotechnology Institute, Nanjing, China). In brief, after 72 h exposure, we collected cells by centrifugation (8000× *g*) at 24 °C for 10 min, and the pellets were homogenized in PBS (pH = 7.2). Then we followed the reagent kit’s detailed procedures. The SOD activity and MDA content were analyzed by measuring the absorbance at 550 nm and 532 nm, respectively, with a microplate reader (Thermo Fisher, Waltham, MA, USA).

### 4.4. Pigment Measurements

Chlorophyll *a* and Carotenoid were determined spectrophotometrically. In brief, after 72 h exposure, the well-mixed cultures were centrifuged at 8000× *g* for 10 min to remove all the supernatant, and the pellets were homogenized with 100% ethanol. Then the mixtures were vigorously shaken with a vortex and placed at −20 °C for 24 h. The extracts were centrifuged at 8000× *g* for 5 min, and the absorbance of the supernatants was evaluated by microplate reader (Thermo Fisher, Waltham, MA, USA). The contents of Chl *a*, Chl *b* and carotenoids were calculated according to Ref. [59], based on their absorbances at 470 nm, 649 nm and 665 nm.

### 4.5. SEM and TEM Measurements

After 72 h TiO_2_ treatment, the cell morphology and surface information were obtained by Scanning Electron Microscope (SEM) (JSM-6330F, JEOL, Tokyo, Japan) following the method of Ref. [60]. In brief, we fixed samples in 2.5% glutaraldehyde for 1 h, then rinsed them with PBS buffer three times to wash out the residual glutaraldehyde. The samples were gradually put in gradient concentrations of ethanol (50%, 70%, 85%, 95% and 100%) to dehydrate the cells and dry them in a critical point dryer with liquid CO_2_. Finally, the samples were coated with gold–palladium and prepared for SEM observations.

After 72 h TiO_2_ treatment, the algal cell ultrastructure was obtained by transmission electron microscope (TEM). In brief, we collected the cells by centrifugation (8000× *g*) at 24 °C for 10 min, and the pellets were fixed in 4% glutaraldehyde overnight at 4 °C. The samples were then washed with PBS (pH = 7.2) by centrifugation (3800× *g*) three times (10 min each time). Samples were then stained with 1% osmium tetroxide for 1 h at 4 °C and washed again with PBS. Samples were successively dehydrated with ethanol, embedded and solidified overnight, then we obtained an ultrathin section by ultra-microtome, and finally the samples were prepared for TEM (JEM-1400, JEOL, Tokyo, Japan) observation.

### 4.6. Statistics Analysis

Statistical analyses were performed using Origin 2018 (OriginLab Corporation, Northampton, MA, USA) and GraphPad Prism 7 (GraphPad Software, San Diego, CA, USA). Data were evaluated by one-way analysis of variance (ANOVA), and Tukey’s honestly significant difference (HSD) test were performed to confirm significant differences between treatments. Statistical significance was set at *p* < 0.05. All data shown in this study are presented as mean ± SD with three or more replicates.

## Figures and Tables

**Table 1:** Effects of three sizes of TiO_2_ particles on the pigment of *S. obliquus* after 72 h exposure. Different letters following values in the same row represent significant difference (Tukey’s HSD, *p* < 0.05). Values are represented as means ± SD (*n* = 3). Chlorophyll *a* (Chl *a*), Chlorophyll *b* (Chl *b*) and Carotenoid (Car) contents are expressed as 10^−13^ g/cell.

Pigment	Control	Bulk TiO_2_	50 nm-TiO_2_	10 nm-TiO_2_
Chl *a*	8.80 ± 2.81 ^a^	8.15 ± 1.16 ^a,b^	3.91 ± 2.06 ^b,c^	3.29 ± 0.16 ^c^
Chl *b*	2.58 ± 0.83 ^a^	2.58 ± 0.24 ^a^	1.79 ± 0.96 ^a^	1.30 ± 0.20 ^a^
Car	6.75 ± 1.85 ^a^	6.43 ± 0.61 ^a^	3.87 ± 1.43 ^a^	3.87 ± 0.28 ^a^
Chl *b*/Chl *a*	0.29 ± 0.01 ^a^	0.32 ± 0.02 ^a^	0.45 ± 0.06 ^b^	0.40 ± 0.08 ^a,b^
Car/Chl *a*	0.78 ± 0.04 ^a^	0.79 ± 0.04 ^a^	1.04 ± 0.14 ^b^	1.17 ± 0.03 ^b^

## Supplementary Materials

The following are available online at https://www.mdpi.com/2223-7747/9/12/1748/s1, Figure S1: Three chlorophyll a fluorescence parameters calculated from induction curves in S. obliquus after 24, 48 and 72 h TiO_2_ treatments.

## References

[1] Binh C.T.T., Peterson C.G., Tong T., Gray K.A., Gaillard J.-F., Kelly J.J. (2015). Comparing Acute Effects of a Nano-TiO_2_ Pigment on Cosmopolitan Freshwater Phototrophic Microbes Using High-Throughput Screening. PLoS ONE.

[2] Weir A., Westerhoff P., Fabricius L., Hristovski K., von Goetz N. (2012). Titanium dioxide nanoparticles in food and personal care products. Environ. Sci. Technol..

[3] Kaegi R., Ulrich A., Sinnet B., Vonbank R., Wichser A., Zuleeg S., Simmler H., Brunner S., Vonmont H., Burkhardt M. (2008). Synthetic TiO_2_ nanoparticle emission from exterior facades into the aquatic environment. Environ. Pollut..

[4] Westerhoff P., Song G., Hristovski K., Kiser M.A. (2011). Occurrence and removal of titanium at full scale wastewater treatment plants: Implications for TiO_2_ nanomaterials. J. Environ. Monit..

[5] Peters R.J.B., van Bemmel G., Milani N.B.L., den Hertog G.C.T., Undas A.K., van der Lee M., Bouwmeester H. (2018). Detection of nanoparticles in Dutch surface waters. Sci. Total Environ..

[6] Xiao B., Zhang Y., Wang X., Chen M., Sun B., Zhang T., Zhu L. (2019). Occurrence and trophic transfer of nanoparticulate Ag and Ti in the natural aquatic food web of Taihu Lake, China. Environ. Sci. Nano.

[7] Zhang M., Yang J., Cai Z., Feng Y., Wang Y., Zhang D., Pan X. (2019). Detection of engineered nanoparticles in aquatic environments: Current status and challenges in enrichment, separation, and analysis. Environ. Sci. Nano.

[8] Kiser M., Westerhoff P., Benn T., Wang Y., Perez-Rivera J., Hristovski K. (2009). Titanium nanomaterial removal and release from wastewater treatment plants. Environ. Sci. Technol..

[9] Gottschalk F., Sun T.Y., Nowack B. (2013). Environmental concentrations of engineered nanomaterials: Review of modeling and analytical studies. Environ. Pollut..

[10] Keller A.A., Lazareva A. (2014). Predicted releases of engineered nanomaterials: From global to regional to local. Environ. Sci. Technol. Lett..

[11] Finkel Z.V., Beardall J., Flynn K., Quigg A., Rees T.A.V., Raven J. (2010). Phytoplankton in a changing world: Cell size and elemental stoichiometry. J. Plankton Res..

[12] Behrenfeld M.J., O’Malley R.T., Siegel D.A., McClain C.R., Sarmiento J.L., Feldman G.C., Milligan A.J., Falkowski P.G., Letelier R.M., Boss E.S. (2006). Climate-driven trends in contemporary ocean productivity. Nature.

[13] Piirsoo K., Pall P., Tuvikene A., Viik M. (2008). Temporal and spatial patterns of phytoplankton in a temperate lowland river (Emajogi, Estonia). J. Plankton Res..

[14] Miller R.J., Bennett S., Keller A.A., Pease S., Lenihan H.S. (2012). TiO_2_ Nanoparticles Are Phototoxic to Marine Phytoplankton. PLoS ONE.

[15] George S., Pokhrel S., Ji Z., Henderson B.L., Xia T., Li L., Zink J.I., Nel A.E., Mädler L. (2011). Role of Fe doping in tuning the band gap of TiO_2_ for the photo-oxidation-induced cytotoxicity paradigm. J. Am. Chem. Soc..

[16] Xiong S., Tang Y., Ng H.S., Zhao X., Jiang Z., Chen Z., Ng K.W., Loo S.C.J. (2013). Specific surface area of titanium dioxide (TiO_2_) particles influences cyto- and photo-toxicity. Toxicology.

[17] von Moos N., Slaveykova V.I. (2014). Oxidative stress induced by inorganic nanoparticles in bacteria and aquatic microalgae--state of the art and knowledge gaps. Nanotoxicology.

[18] Gervais F., Siedel U., Heilmann B., Weithoff G., Heisig-gunkel G., Nicklisch A. (2003). Small-scale vertical distribution of phytoplankton, nutrients and sulphide below the oxycline of a mesotrophic lake. J. Plankton Res..

[19] Descy J.-P., Hardy M.-A., Sténuite S., Pirlot S., Leporcq B., Kimirei I., Sekadende B., Mwaitega S.R., Sinyenza D. (2005). Phytoplankton pigments and community composition in Lake Tanganyika. Freshw. Biol..

[20] Williamson C.E., Stemberger R.S., Morris D.P., Frost T.M., Paulsen S.G. (1996). Ultraviolet radiation in North American lakes: Attenuation estimates from DOC measurements and implications for plankton communities. Limnol. Oceanogr..

[21] Zhang Y., Yin Y., Zhang E., Zhu G., Liu M., Feng L., Qin B., Liu X. (2011). Spectral attenuation of ultraviolet and visible radiation in lakes in the Yunnan Plateau, and the middle and lower reaches of the Yangtze River, China. Photochem. Photobiol. Sci..

[22] Li F., Liang Z., Zheng X., Zhao W., Wu M., Wang Z. (2015). Toxicity of nano-TiO_2_ on algae and the site of reactive oxygen species production. Aquat. Toxicol..

[23] Li M., Chen D., Liu Y., Chuang C.Y., Kong F., Harrison P.J., Zhu X., Jiang Y. (2018). Exposure of engineered nanoparticles to *Alexandrium tamarense* (Dinophyceae): Healthy impacts of nanoparticles via toxin-producing dinoflagellate. Sci. Total Environ..

[24] Sendra M., Moreno-Garrido I., Yeste M.P., Gatica J.M., Blasco J. (2017). Toxicity of TiO_2_, in nanoparticle or bulk form to freshwater and marine microalgae under visible light and UV-A radiation. Environ. Pollut..

[25] Planchon M., Jittawuttipoka T., Cassier-Chauvat C., Guyot F., Gelabert A., Benedetti M.F., Chauvat F., Spalla O. (2013). Exopolysaccharides protect *Synechocystis* against the deleterious effects of titanium dioxide nanoparticles in natural and artificial waters. J. Colloid Interface Sci..

[26] Roy B., Chandrasekaran H., Palamadai Krishnan S., Chandrasekaran N., Mukherjee A. (2018). UVA pre-irradiation to P25 titanium dioxide nanoparticles enhanced its toxicity towards freshwater algae *Scenedesmus obliquus*. Environ. Sci. Pollut. Res..

[27] Pospíšil P. (2016). Production of reactive oxygen species by photosystem II as a response to light and temperature stress. Front Plant Sci..

[28] Vass I. (2011). Role of charge recombination processes in photodamage and photoprotection of the photosystem II complex. Physiol. Plant.

[29] D’Alessandro S., Havaux M. (2019). Sensing β-carotene oxidation in photosystem II to master plant stress tolerance. New Phytol..

[30] Sharma P., Jha A.B., Dubey R.S., Pessarakli M. (2012). Reactive oxygen species, oxidative damage, and antioxidative defense mechanism in plants under stressful conditions. J. Bot..

[31] Foyer C.H., Ruban A.V., Noctor G. (2017). Viewing oxidative stress through the lens of oxidative signalling rather than damage. Biochem. J..

[32] Chalifour A., Juneau P. (2011). Temperature-dependent sensitivity of growth and photosynthesis of *Scenedesmus obliquus*, *Navicula pelliculosa* and two strains of *Microcystis aeruginosa* to the herbicide atrazine. Aquat. Toxicol..

[33] Roy R., Parashar A., Bhuvaneshwari M., Chandrasekaran N., Mukherjee A. (2016). Differential effects of P25 TiO_2_ nanoparticles on freshwater green microalgae: *Chlorella* and *Scenedesmus* species. Aquat. Toxicol..

[34] Aruoja V., Dubourguier H.-C., Kasemets K., Kahru A. (2009). Toxicity of nanoparticles of CuO, ZnO and TiO_2_ to microalgae *Pseudokirchneriella subcapitata*. Sci. Total. Environ..

[35] Hartmann N.B., Von der Kammer F., Hofmann T., Baalousha M., Ottofuelling S., Baun A. (2010). Algal testing of titanium dioxide nanoparticles—Testing considerations, inhibitory effects and modification of cadmium bioavailability. Toxicol. Potential Hazard Nanopart. Prop. Biol. Environ. Eff..

[36] Kulacki K.J., Cardinale B.J. (2012). Effects of nano-titanium dioxide on freshwater algal population dynamics. PLoS ONE.

[37] Lee W.-M., An Y.-J. (2013). Effects of zinc oxide and titanium dioxide nanoparticles on green algae under visible, UVA, and UVB irradiations: No evidence of enhanced algal toxicity under UV pre-irradiation. Chemosphere.

[38] Fu L., Hamzeh M., Dodard S., Zhao Y.H., Sunahara G.I. (2015). Effects of TiO_2_ nanoparticles on ROS production and growth inhibition using freshwater green algae pre-exposed to UV irradiation. Environ. Toxicol. Pharmacol..

[39] Xu K., Juneau P. (2016). Different physiological and photosynthetic responses of three cyanobacterial strains to light and zinc. Aquat. Toxicol..

[40] Xu K., Jiang H., Juneau P., Qiu B. (2012). Comparative studies on the photosynthetic responses of three freshwater phytoplankton species to temperature and light regimes. J. Appl. Phycol..

[41] Deblois C.P., Juneau P. (2012). Comparison of resistance to light stress in toxic and non-toxic strains of *Microcystis aeruginosa* (Cyanophyta). J. Phycol..

[42] Williamson C.J., Cook J., Tedstone A., Yallop M., McCutcheon J., Poniecka E., Campbell D., Irvine-Fynn T., McQuaid J., Tranter M. (2020). Algal photophysiology drives darkening and melt of the Greenland Ice Sheet. PNAS.

[43] Juneau P., Berdey A.E., Popovic R. (2002). PAM fluorometry in the determination of the sensitivity of Chlorella vulgaris, Selenastrum capricornutum, and Chlamydomonas reinhardtii to copper. Arch. Environ. Contam. Toxic..

[44] Unnep R., Paul S., Zsiros O., Kovacs L., Szekely N., Steinbach G., Appavou M., Porcar L., Holzwarth A.R., Garab G. (2020). Thylakoid membrane reorganizations revealed by small-angle neutron scattering of Monstera deliciosa leaves associated with non-photochemical quenching. Open Biol..

[45] Xu K., Racine F., He Z., Juneau P. (2019). Impacts of hydroxyphenylpyruvate dioxygenase (HPPD) inhibitor (mesotrione) on photosynthetic processes in *Chlamydomonas reinhardtii*. Environ. Pollut..

[46] Müller P., Li X.-P., Niyogi K.K. (2001). Non-Photochemical Quenching. A Response to Excess Light Energy. Plant Physiol..

[47] Rocchetta I., Küpper H. (2009). Chromium- and copper-induced inhibition of photosynthesis in *Euglena gracilis* analysed on the single-cell level by fluorescence kinetic microscopy. New Phytol..

[48] Li J., Schiavo S., Rametta G., Miglietta M.L., Ferrara V.L., Wu C., Manzo S. (2017). Comparative toxicity of nano ZnO and bulk towards marine algae *Tetraselmis suecica* and *Phaeodactylum tricornutum*. Environ. Sci. Pollut. Res..

[49] Middepogu A., Hou J., Gao X., Lin D. (2018). Effect and mechanism of TiO_2_ nanoparticles on the photosynthesis of *Chlorella pyrenoidosa*. Ecotoxicol. Environ. Saf..

[50] Shull T., Kurepa J., Smalle J. (2019). Anatase TiO_2_ Nanoparticles induce autophagy and chloroplast degradation in Thale Cress (*Arabidopsis thaliana*). Environ. Sci. Technol..

[51] Mendoza-Cózatl D.G., Jobe T.O., Hauser F., Schroeder J.I. (2011). Long-distance transport, vacuolar sequestration and transcriptional responses induced by cadmium and arsenic. Curr. Opin. Plant Biol..

[52] Priyadarshini E., Priyadarshini S.S., Pradhan N. (2019). Heavy metal resistance in algae and its application for metal nanoparticle synthesis. Appl. Microbiol. Biotechnol..

[53] Wilson N. (2018). Nanoparticles: Environmental problems or problem solvers?. Bioscience.

[54] Stanier R.Y., Kunisawa M.M., Cohen-Bazire G. (1971). Purification and properties of unicellular blue-green algae (order Chroococcales). Bacteriol. Rev..

[55] Xu K., Zhengke L., Liu S.-W., Qiu B.-S. (2017). Effects of iron deficiency on the growth and photosynthesis of three bloom-forming cyanobacterial species isolated from Lake Taihu: Cyanobacteria and iron deficiency. Phycol. Res..

[56] Kitajima M., Butler W. (1975). Quenching of chlorophyll fluorescence and primary photochemistry in chloroplasts by dibromo-thymoqu. Biochim. Biophys. Acta.

[57] Genty B., Briantais J.M., Baker N.R. (1989). The relationship between the quantum yield of photosynthetic electron transport and quenching of chlorophyll fluorescence. Biochim. Biophys. Acta Gen. Subj..

[58] Bilger W., Bjorkman O. (1990). Role of the xanthophyll cycle in photoprotection elucidated by measurements of light-induced absorbance changes, fluorescence and photosynthesis in leaves of *Hedera canariensis*. Photosynth. Res..

[59] Rowan K.S. (1989). Photosynthetic Pigments of Algae.

[60] Zhang S., Deng R., Lin D., Wu F. (2017). Distinct toxic interactions of TiO_2_ nanoparticles with four coexisting organochlorine contaminants on algae. Nanotoxicology.

