# Supplementary material for: Effects of Titanium Dioxide Nanoparticles on Photosynthetic and Antioxidative Processes of Scenedesmus obliquus
Source: Plants (Basel). 2020 Dec 10;9(12):1748. doi: 10.3390/plants9121748 (PMC7763043; doi:10.3390/plants9121748)
Supplement: Supplementary file 1 [file plants-09-01748-s001.pdf]

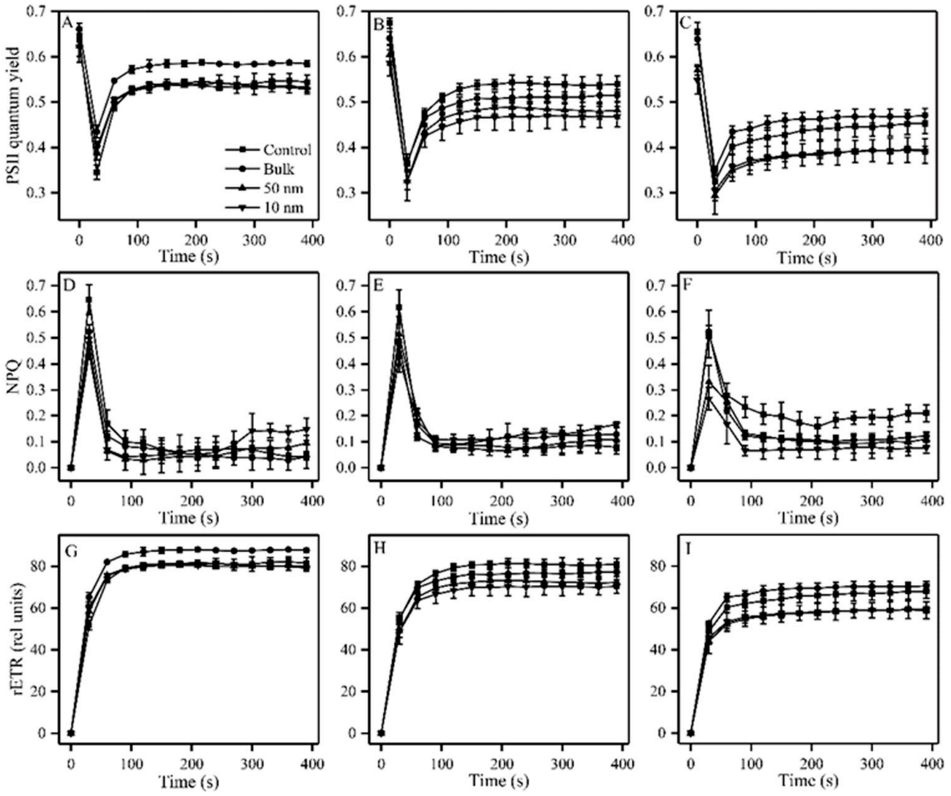

Figure S1. Three chlorophyll *a* fluorescence parameters calculated from induction curves in *S. obliquus* after 24, 48 and 72 h TiO_2 treatments. (**A**) PSII quantum yield after 24 h treatment; (**B**) PSII quantum yield after 48 h treatment; (**C**) PSII quantum yield after 72 h treatment; (**D**) NPQ after 24 h treatment; (**E**) NPQ after 48 h treatment; (**F**) NPQ after 72 h treatment; (**G**) rETR after 24 h treatment; (H) rETR after 48 h treatment; (**I**) rETR after 72 h treatment.
